# The Structure of the Spinal Cord Ependymal Region in Adult Humans Is a Distinctive Trait among Mammals

**DOI:** 10.3390/cells10092235

**Published:** 2021-08-28

**Authors:** Alejandro Torrillas de la Cal, Beatriz Paniagua-Torija, Angel Arevalo-Martin, Christopher Guy Faulkes, Antonio Jesús Jiménez, Isidre Ferrer, Eduardo Molina-Holgado, Daniel Garcia-Ovejero

**Affiliations:** 1 Laboratory of Neuroinflammation, Hospital Nacional de Paraplejicos, 45071 Toledo, Spain; alejandrotorrillas13@gmail.com (A.T.d.l.C.); bpaniagua@externas.sescam.jccm.es (B.P.-T.); aarevalom@sescam.jccm.es (A.A.-M.); eduardom@sescam.jccm.es (E.M.-H.); 2 School of Biological & Chemical Sciences, Queen Mary University of London, London E1 4NS, UK; c.g.faulkes@qmul.ac.uk; 3 Departamento de Biología Celular, Genética y Fisiología, Universidad de Málaga, Campus de Teatinos, 29071 Malaga, Spain; ajjimenez@uma.es; 4 Instituto de Investigación Biomédica de Málaga (IBIMA), 29010 Malaga, Spain; 5 Institut de Neuropatologia, Servei d’Anatomia Patològica, IDIBELL-Hospital Universitari de Bellvitge, Universitat de Barcelona, 08908 L’Hospitalet de Llobregat, Spain; 8082ifa@gmail.com

**Keywords:** ependymal cell, epithelial to mesenchymal transition, neural stem cells, spinal cord injury, repair

## Abstract

In species that regenerate the injured spinal cord, the ependymal region is a source of new cells and a prominent coordinator of regeneration. In mammals, cells at the ependymal region proliferate in normal conditions and react after injury, but in humans, the central canal is lost in the majority of individuals from early childhood. It is replaced by a structure that does not proliferate after damage and is formed by large accumulations of ependymal cells, strong astrogliosis and perivascular pseudo-rosettes. We inform here of two additional mammals that lose the central canal during their lifetime: the Naked Mole-Rat (NMR, *Heterocephalus glaber*) and the mutant hyh (*hydrocephalus with hop gait*) mice. The morphological study of their spinal cords shows that the tissue substituting the central canal is not similar to that found in humans. In both NMR and hyh mice, the central canal is replaced by tissue reminiscent of normal lamina X and may include small groups of ependymal cells in the midline, partially resembling specific domains of the former canal. However, no features of the adult human ependymal remnant are found, suggesting that this structure is a specific human trait. In order to shed some more light on the mechanism of human central canal closure, we provide new data suggesting that canal patency is lost by delamination of the ependymal epithelium, in a process that includes apical polarity loss and the expression of signaling mediators involved in epithelial to mesenchymal transitions.

## 1. Introduction

**Figure 1:**
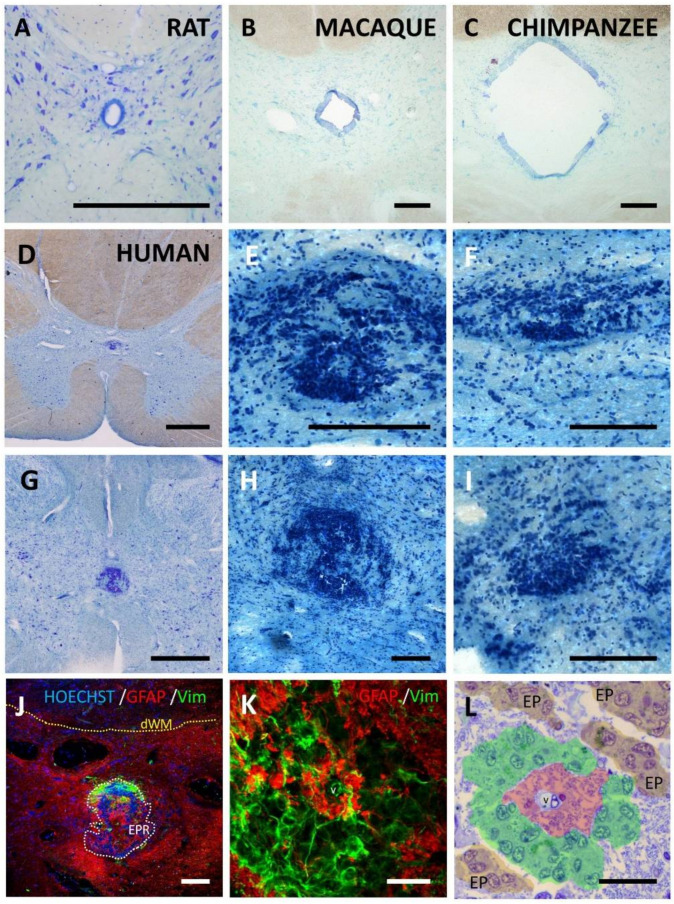
Ependymal region organization in different mammal species. In toluidine blue-stained sections, ependymal lining surrounding a patent central canal can be observed in most mammals such as rat (**A**), macaque (**B**) or chimpanzee (**C**). (**D**) Young and adult humans, on the contrary, mostly show absence of central canal which is substituted by a new organization of the ependymal region that includes large accumulation of cells (**D**–**I**). Examples from different individuals are shown: (**D**) male, 52 years *(A10/017 sample)*; (**E**,**F**) higher magnification details of different spinal levels of (**D**); (**G**) male 39 years *(A10/044 sample)*; (**H**) higher magnification detail of G; (**I**) Male 47 years *(A10/067 sample)*. (**J**) In adult humans, the new structure in the ependymal region (EPR) substituting former canal also includes strong astrogliosis (GFAP immunoreactivity, red) and the presence of (**K**,**L**) perivascular pseudorosettes, i.e., cells expressing vimentin (green) radially oriented around a central vessel (v), separated from it by a hypocellular GFAP^+^ region. Pseudocolors in L highlight the GFAP hypocellular region (red) that surrounds the vessel (v), cells radially oriented (green) and ependymal cell groups around the perivascular pseudorosette (EP). dWM, dorsal white matter; Vim, vimentin. Magnification bars: (**A**), 250 µm; (**B**,**C**,**E**,**F**,**H**–**J**), 200 µm; (**D**,**G**), 1 mm; (**K**,**L**), 25 µm.

The ependymal region is a crucial instructor of repair in species that spontaneously regenerate the damaged spinal cord, such as fish or salamanders [1,2]. It proliferates after injury and coordinates the reparative process [3]. In non-regenerating animals, such as mammals, this region is also organized as a patent central canal surrounded by cells comprising a variety of ependymal subtypes and other accompanying cells [4,5,6,7,8,9,10,11], and reacts to injury by increasing proliferation and giving rise to ependymal cells and, occasionally, astrocytes [12,13,14,15,16]. Although it is known that the mammalian ependymal region shows specific features in different domains (dorsal/ventral/lateral) [7,8,9], between rostral–caudal regions [17] and particularities related to species [6,18,19,20] or age [4,21,22,23], most mammals share a common pattern of organization, from early ages to adulthood and aging (Figure 1A–C). A thorough literature search shows that this pattern can be found in species phylogenetically close to humans (primates: marmoset [24]; macaque; chimpanzee, Figure 1B,C), in large mammals (cow [25]; giraffe [26]; pig [27]; dog [28]; cat [29]; sheep [30]), in small–medium size mammals (rat/mouse, Figure 1A; ferret [31]; hedgehog [32]) and in aquatic (sea lion [33]) or flying (fruit bat [34]) mammals.

However, a different organization of the ependymal region is found in adult humans (Figure 1D–L). During childhood, it is similar to other mammals in structure and expression of some ependymal markers [5,35,36,37,38], but during late infancy to adulthood, the central canal is lost in the majority of individuals being substituted by a structure largely different from the rest of the species [20,39,40,41]. After the central canal is lost, it is substituted by large accumulations of ependymal cells not enclosing a lumen (Figure 1D–I), intense astrogliosis (Figure 1J,K) and perivascular pseudorosettes (Figure 1L) [5,20] and does not proliferate in response to spinal cord injury [42]. The expression of other markers related to neural stem cells can be found in [5,6,7,19,20,35,36].

The trigger that makes humans lose the central canal after childhood, the mechanism by which this process develops and the consequences in the physiology of the spinal cord and its response to injury are still unknown. The study of this phenomenon is rather complex due to the paucity of human spinal cord samples available and the lack of animal models. In the search for a natural model, we found two candidates: the Naked Mole-Rat (NMR, *Heterocephalus glaber*) and the hyh (*hydrocephalus with hop gait*) mutant mouse [43]. The ependymal region of the Naked Mole-Rats has not been described to date, but the observation of images from their spinal cord in other publications suggested us that they might present no patent canal [44,45]. The hyh mice carry a hypomorphic missense mutation in the NAPA gene encoding soluble N-ethylmaleimide- sensitive factor (NSF) attachment protein alpha (SNAP-alpha) [43,46,47]. These mice have been shown to lose central canal during late embryonic stages [48], although this observation has not been further extended ever since.

In the current work, we confirm (and in the case of NMR we report for the first time) that adult individuals of these animals lack a central canal. However, we show that in both cases the structure substituting the former canal is largely different from that found in adult humans, not including large accumulations of cells, neither gliosis nor perivascular pseudorosettes. In addition, we provide new data on the possible mechanism of central canal closure in humans by studying human spinal cord postmortem samples with a partially patent canal.

## 2. Material and Methods

### 2.1. Animals

#### 2.1.1. Naked Mole-Rats

Naked Mole-Rats (NMR, *Heterocephalus glaber*) were bred at Queen Mary University of London and housed in their natal colonies as previously described (e.g., [49]). In this study, we used one non-breeding female (2 years of age) and 5 non-breeding males (ages: 2, 9 and 10 years). Non-reproductive status was verified behaviorally, morphologically and/or by examination of the reproductive tract as previously described [49,50]. The research was conducted in accordance with the U.K. Home Office Animals (Scientific Procedures) Act 1986. Because tissue sample collection was post-euthanasia, additional local ethical approval for NMR work was not required for this study. For the immunohistochemical study, animals were terminally anesthetized with sodium pentobarbitone (Pentoject; Animalcare, Ltd., New York, United Kingdom; 50 mg/kg) and perfused transcardially with phosphate-buffered saline (PBS), followed by 4% paraformaldehyde in 0.1 M phosphate buffer (pH 7.4). The spinal cords were postfixed overnight in the same fixative, and maintained in PBS. Spinal cords were then extracted and cervical, thoracic or lumbar fragments of the spinal cord (5 mm in length) were embedded in low melting agarose with 10% sucrose in 0.1 M PB. Serial transverse vibrating microtome sections (40 µm) were obtained and stored in Olmos solution at −18 °C until use.

#### 2.1.2. Hyh Mice

Mutant hyh mice (*hydrocephalus with hop gait*, B6C3Fe-a/a-hyh/J strain) [43] carry a point mutation in the Napa gene that encodes α-Snap [46,47], a protein involved in membrane fusion events. Mice were obtained from The Jackson Laboratory (Bar Harbor, ME, USA) and bred at the Animal Experimentation Service of the University of Malaga. The housing, handling, care and processing of the animals were conducted in accordance with the European and Spanish laws (DC 86/609/CEE and RD 1201/2005, 2010/63/ EU) and was approved by the Institutional Animal Care and Use Committee of the University of Malaga, Spain (CEUMA) and the Regional Government Council (Junta de Andalucía, Spain) (protocol # 4-2015-A). Mutant hyh mice were identified by clinical inspection and genotyping [51]. The animals used in this study (5 males) were anesthetized with intraperitoneally administered Dolethal (sodium pentobarbital; Vétoquinol, Lure, France; 0.2 mg/g bodyweight) and intracardially perfused with 4% paraformaldehyde at 20 days after birth (postnatal age 20, P20). Spinal cords were extracted and cervical, thoracic or lumbar fragments of the spinal cord (5 mm in length) were embedded in low melting agarose with 10% sucrose in 0.1 M PB. Serial transverse vibrating microtome sections (40 µm) were obtained and stored in Olmos solution at −18 °C until use.

#### 2.1.3. Chimpanzee

Postmortem chimpanzee spinal cord samples were kindly provided by Dr. Eva Martinez Nevado (Zoo-Aquarium, Madrid, Spain). A 25 year old female chimpanzee (*Pan troglodites*) from the Zoo inhouse colony, who was being treated for heart disease (cardiomyopathy, left apex thrombus and tricuspid valve insufficiency), worsened after anesthesia during health checking and finally died after 48 h. During autopsy, a 10 mm spinal cord block was obtained from low cervical/high thoracic levels and immersed in formalin for 3 days. Spinal block was then rinsed in PBS and serial transverse vibrating microtome sections (40 µm) were obtained and stored at −18 °C in Olmos solution until use.

#### 2.1.4. Macaque

Macaque tissue was gently provided by Drs. Javier Cudeiro, Casto Rivadulla (NEUROcom, School of Health Sciences University of A Coruña, A Coruña, Spain) and Juan Aguilar (Hospital Nacional de Paraplejicos). Spinal cord was extracted postmortem from a 7 year old male macaque monkey *(Macaca mulatta)* used in previous studies [52]. All procedures followed the rules of the Physiological Spanish Society, the International Council for Laboratory Animal Science, and the European Union (No. 2010/63/EU) and were approved by the ethics committee for animal research of the University Hospital of A Coruña. The animal was intracardially perfused with 4% paraformaldehyde and postfixed in the same fixative for 3 days. Spinal cord was extracted and divided in 5 mm blocks. Serial transverse vibrating microtome sections (40 µm) were obtained and stored at −18 °C in Olmos solution until use.

### 2.2. Human Tissue

Human tissue was obtained from two public tissue biobanks: the HUFA BioBank (Biobanco del Hospital Universitario Fundación Alcorcón, Alcorcón, Spain) and the Neurological Tissue Bank (Banco de Tejidos Neurológicos, IDIBELL- Hospital Universitario de Bellvitge, Hospitalet de Llobregat, Barcelona, Spain). Tissue was provided in 5–8 mm thick formalin fixed blocks or vibratome free floating sections. Tissue donation always included written informed consent from donors while alive or from their families after death. Data from donors and handling of samples obtained from all the Biobanks included in this study were processed after approval by the Clinical Research Ethics Committee (CEIC) in Toledo (Spain), in accordance with Spanish law and International Guidelines (LOPD15/1999; RD 1720/2007; Declaration of Helsinki, 2008). Samples were obtained from ten deceased individuals without clinical or histopathological involvement of the spinal cord (Table 1). Upon receipt, tissue blocks were embedded in low melting agarose with 10% sucrose in 0.1 M PBS and cut into serial transverse sections (40 µm) with a vibrating microtome (Leica VT 1000 M). Sections were then stored at −18 °C in Olmos solution until use.

### 2.3. Histology and Immunohistochemistry

#### 2.3.1. Nissl Staining

Free floating sections were mounted on gelatin covered slides and dried overnight. After two rinses with distilled water, slices were stained with 0.01% toluidine blue (Merck, Madrid, Spain) in 0.2 M Walpole buffer for 20 min, dehydrated in graded ethanol solutions, cleared with xylene and covered with DPX mounting medium (VWR, Barcelona, Spain).

#### 2.3.2. Immunohistochemistry

Immunohistochemistry was performed on sections rinsed in rinse solution (RS) containing 0.1 M phosphate-buffered saline (pH 7.4), 0.3% Triton X-100 and 0.3% bovine serum albumin (BSA). An additional methanol pretreatment (50% methanol 1 min, followed by 100% methanol 9 min) was used for better detection of transcription factors and cytoskeletal proteins, when needed. Only for human samples, sections were subjected to antigen unmasking, consisting of a 30 min pretreatment with a 0.05% solution of citraconic anhydride (#27430, Sigma-Aldrich, St. Louis, MO, USA) at 96 °C, followed by temperature re-accommodation at room temperature for at least 30 additional minutes [53,54]. The sections were then incubated for 2 nights with the primary antibodies (Table 2) diluted in RS with 0.3% Triton X-100 and 5% normal goat serum, or 5% BSA in cases where the antiserum was generated in goat.

After extensive rinsing, the primary antibodies were detected using fluorophore-conjugated antibodies (Table 3). Some antibodies (phospho Smad3, SNAI1, activated Notch-NICD) required an intermediate incubation with biotinylated secondary antibodies followed by incubation with Alexa conjugated streptavidin (Table 3). Nuclear counterstaining with bisbenzimide was performed (BBZ, Hoechst 33258 pentahydrate, Invitrogen, Waltham, MA, USA; 1:5000). Sections were mounted with Immumount (Thermo Fisher, Waltham, MA, USA) and analyzed with a LEICA SP5 confocal microscope at the Microscopy Facility in the National Hospital for Paraplegics (Toledo, SESCAM). Images were transferred to ImageJ (NIH, Bethesda, MD, USA) for cropping; they were adjusted to optimize contrast and brightness. Noise was reduced using a median filter with Fiji (http://pacific.mpi-cbg.de), a scientific image processing application based on ImageJ (http://rsb.info.nih.gov/ij). Control experiments were performed to rule out the interference of non-specific staining in parallel with the complete assays. The incubation of tissue without primary antibodies, with all other steps being identical to those described above, eliminated all staining, with the exception of some blood cells and lipofuscin autofluorescence present mostly in human tissue.

### 2.4. Transmission Electron Microscopy

A series of tissue slices were immersed in a fixative containing 2% paraformaldehyde and 2.5% glutaraldehyde in 0.1 M PB for 5 days. Sections were then rinsed in 0.1 M PB, postfixed in 1% osmium tetroxide in 0.1 M PB with 5% glucose, dehydrated in acetone, and embedded in Araldite resin (Araldite 502 Kit, #13900, Electron Microscopy Sciences, Hatfield, PA, USA). Semithin sections (2 µm) were obtained with a Leica EM UC6 Ultramicrotome and stained with 1% toluidine blue in 1% borax. For transmission electron microscopy, semithin sections were re-embedded, cut in ultrathin (65 nm) sections and mounted on formvar-coated grids as described before [9]. Grids were stained with uranyl acetate (#22400, Electron Microscopy Sciences) and then with lead citrate (#17810 Electron Microscopy Sciences) according to the protocol of Venable and Coggeshall [55]. Analysis of the samples was performed with a Jeol 1200EXII electron microscope at the Electron Microscopy Facility in the Cajal Institute (CSIC, Madrid, Spain).

## 3. Results

### 3.1. Adult Naked Mole-Rats (Heterocephalus Glaber) and the Hyh (Hydrocephalus with Hop Gait) Mutant Mice Lack Central Canal, but the Substituting Structure Is Notably Different from That in Humans

#### 3.1.1. Naked Mole-Rats

**Figure 2:**
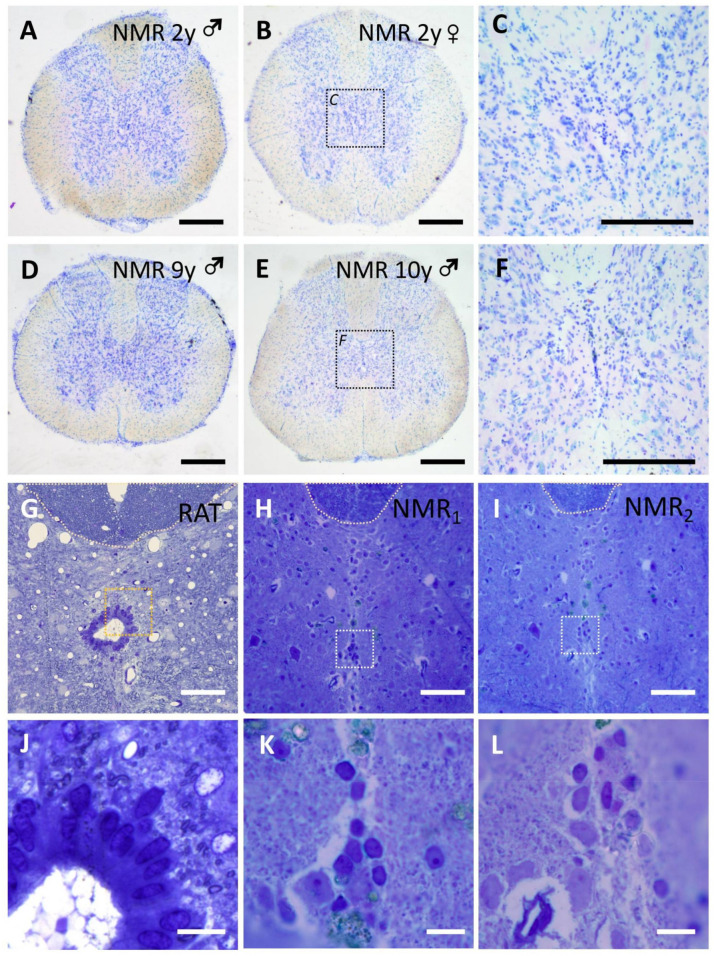
Central canal is absent in adult Naked Mole-Rats (NMR). Toluidine blue stained sections of adult NMR show an even distribution of cell nuclei in lamina X in adult and mature NMR. (**A**) Male (♂) 2 years old; (**B**) female (♀) 2 years old; (**C**) higher magnification of dashed square shown in B; (**D**) 9 y old male; (**E**) 10y old male; (**F**) higher magnification of dashed square shown in E. (**G**–**L**) Toluidine blue-stained semithin sections showing the normal appearance of the ependymal region in rats (**G**), compared with spinal cords from NMR (**H**,**I**) in which small accumulations of cell nuclei can be found dorsoventrally oriented in the midline, but no central canal is found. Dashed squares delimitate the higher magnification details shown in (**J–L**). Magnification bars: (**A**,**B**,**D**,**E**), 500 µm; (**C**,**F**), 250 µm; (**G**–**I**), 50 µm; (**J**–**L**), 10 µm.

**Figure 3:**
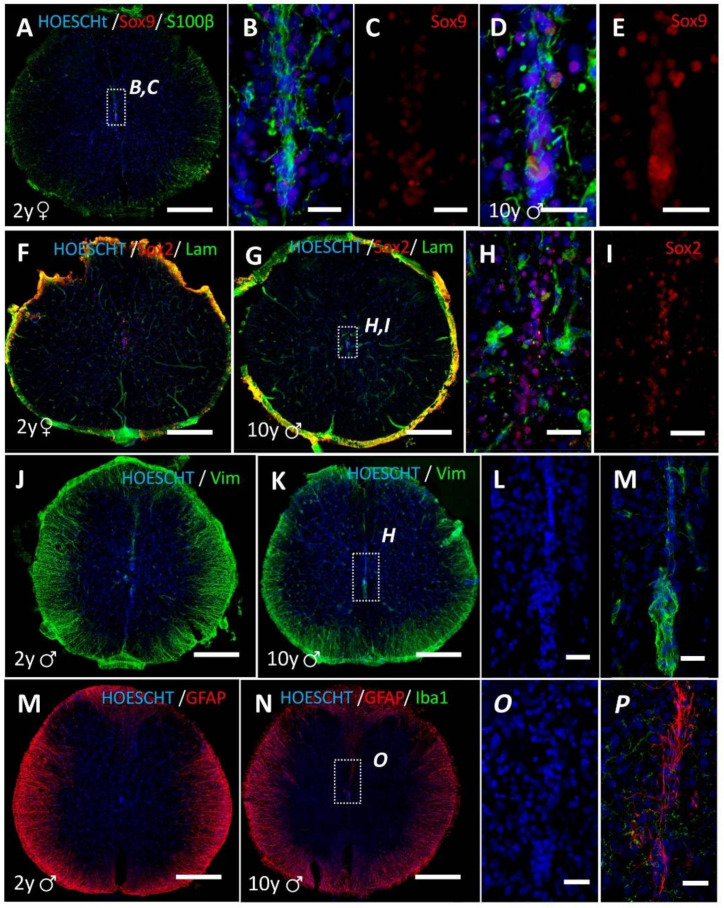
Histological features of the ependymal region in adult NMR. (**A**) Dorsoventral accumulations of cells in the midline express Sox9 (red) and S100β (green) proteins. Higher magnification of the dashed square region is shown in (**B**,**C**). (**D**,**E**) Same features can be observed in older (10 years old) individuals. (**F**–**I**) the ependymal remnant in NMR also show expression of Sox2 (green) and a normal distribution of blood vessels (laminin, green) both in 2 years and older individuals. (**J**–**M**) Midline accumulation of cells in NMR also expresses long vimentin immunoreactive processes (green). (**M**) The ependymal region of adult NMR does not show strong GFAP-ir gliosis, such as in humans. Strong GFAP signal is found in white matter astrocytes, but not in gray matter or the lamina X. (**N**,**O**) GFAP is found in the ependymal remnant of older NMR individuals (9–10 years old), not forming a widespread gliosis but depicting long processes of midline cells dorsoventrally oriented. This GFAP expression is probably not due to astrocyte response to inflammatory cues, since microglial morphology in the surroundings (Iba1, green) show non-activated morphologies (**P**). ♂, male; ♀, female. Magnification bars: (**A**,**F**,**G**,**J**,**K**,**M**,**N**), 500 µm; (**B**–**E**,**H**,**I**,**L**,**M**,**O**,**P**), 50 µm.

All the individuals studied showed age- and sex-independent absence of central canal in their spinal cords (Figure 2). In the central gray, at the position where the central canal is usually found in other species, the appearance of neural parenchyma is similar to that observed in the rest of the lamina X, and similar to the appearance of periependymal areas in other species (Figure 2), but does not show large cellular accumulations such as those observed in humans. Only small clusters of ependymal-like nuclei dorsoventrally oriented in the midline seems to resemble a remnant of the former ependymal region. These cells express Sox2 and Sox9 (Figure 3A–D,F–I), and some of them also vimentin (Figure 3J–M) or the ependymal/astrocyte marker S100β (Figure 3A,B,D).

Unlike humans, no astrogliosis was observed in the central gray matter nor in the whole lamina X. In the central gray matter of NMR, GFAP expression was equivalent to that found in the normal parenchyma (Figure 3M–P). Among the small ependymal-like clusters of the midline, GFAP was generally absent or scarcely expressed (Figure 3M), but in older individuals (9–10 years old) strong GFAP immunoreactivity in some midline cells with long GFAP^+^ processes can be found (Figure 3N–P). Microglial cells surrounding these GFAP^+^ cells show non-activated morphologies (Iba1^+^ cells, Figure 3N,P).

Finally, no perivascular pseudo-rosettes were found in the NMR ependymal remnant, unlike in humans. In the NMR, the distribution and density of vasculature showed no apparent abnormalities when stained with laminin (Figure 3F–H).

#### 3.1.2. Hyh Mice

**Figure 4:**
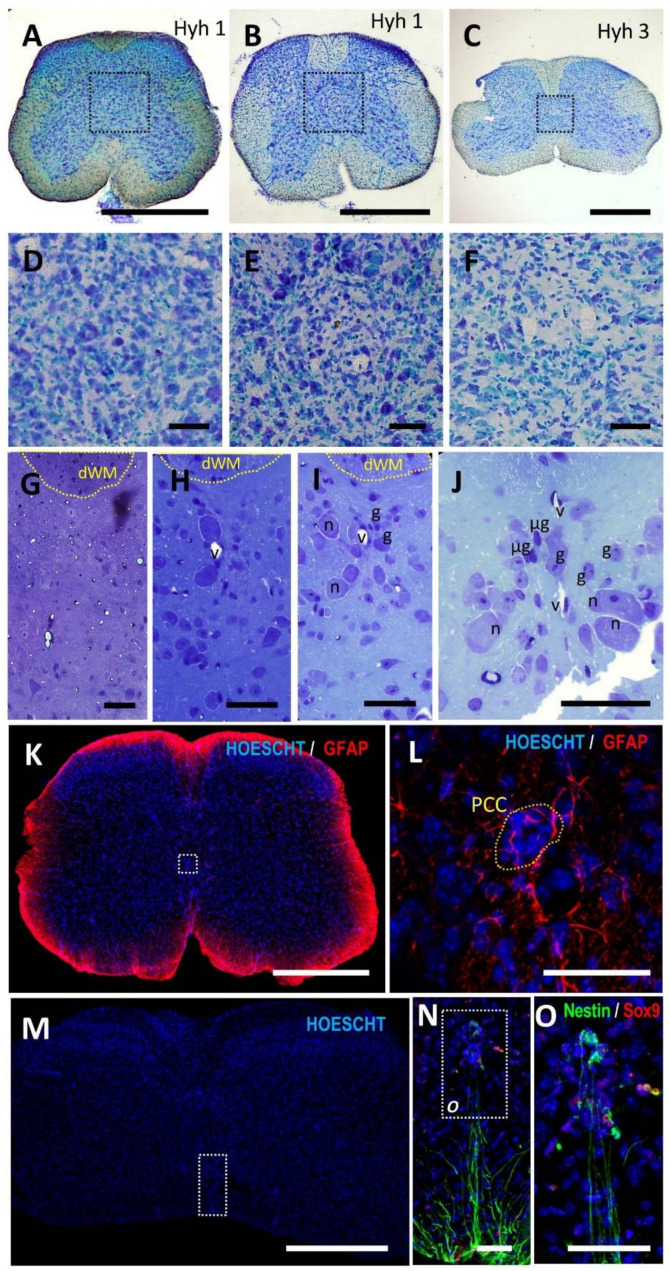
Histological features of the ependymal region in adult hyh mice. (**A**–**C**) Toluidine blue stained sections of adult hyh show the absence of central canal cell nuclei in lamina X in three different individuals. Higher magnification pictures of the squared regions are showed in (**D**–**F**). (**G**–**J**) Toluidine blue-stained semithin sections show the distribution of cells in the central gray matter, in which vessels (v), neurons (n), microglial (µg) and other glial (g) cell nuclei can be observed without forming dorsoventral alignments such as those found in NMR. (**K**) GFAP immunostaining (red) show absence of gliosis in the gray matter. (**L**) occasional pseudocanal-like structures (PCC), i.e., cells enclosing a lumen, can be found in the central gray of hyh mice. (**M**–**O**) Details of the ependymal remnant in hyh mice (indicated by dashed rectangles) showing cells expressing Sox9 (red) and Nestin (green). Magnification bars: (**A**–**C**,**K**,**M**), 500 µm; (**D**–**J**,**L**,**N**,**O**), 50 µm.

All the individuals studied showed absence of the central canal (Figure 4). Instead of a well-defined ependymal region surrounding it, cells were evenly distributed, not forming clusters or large accumulations such as those observed in adult humans. The observation of cell nuclei in the central gray midline using toluidine blue stained semithin sections showed morphologies resembling microglia, other glia and neurons, as well as small capillaries enclosed by endothelial cells (Figure 4G–J). Dorsoventrally oriented cell alignments such as those observed in NMR were not clearly distinguished. On the other hand, small groups of ependymal cells enclosing small cavities were occasionally found (Figure 4L,O), forming pseudocanal-like structures. Cells at these structures express Sox9 and a small part, also nestin (Figure 4N,O).

As in NMR, astrocytosis was not found in the central gray matter of hyh mice (Figure 4K). GFAP expression followed the general distribution observed in many species: high expression in the outer rim of the spinal cord, including glia limitans, and normal astrocytic expression in the parenchyma, with no reactive morphology, including those in the central gray (Figure 4K,L).

Distribution of vessels and capillaries showed a normal appearance and no perivascular pseudorosettes were found unlike in humans (Figure 4G–J).

### 3.2. The Closure of the Central Canal in the Adult Human Ependymal Region Is Preceded by a Delamination That Shows Features of Epithelial to Mesenchymal Transition (EMT)

**Figure 5:**
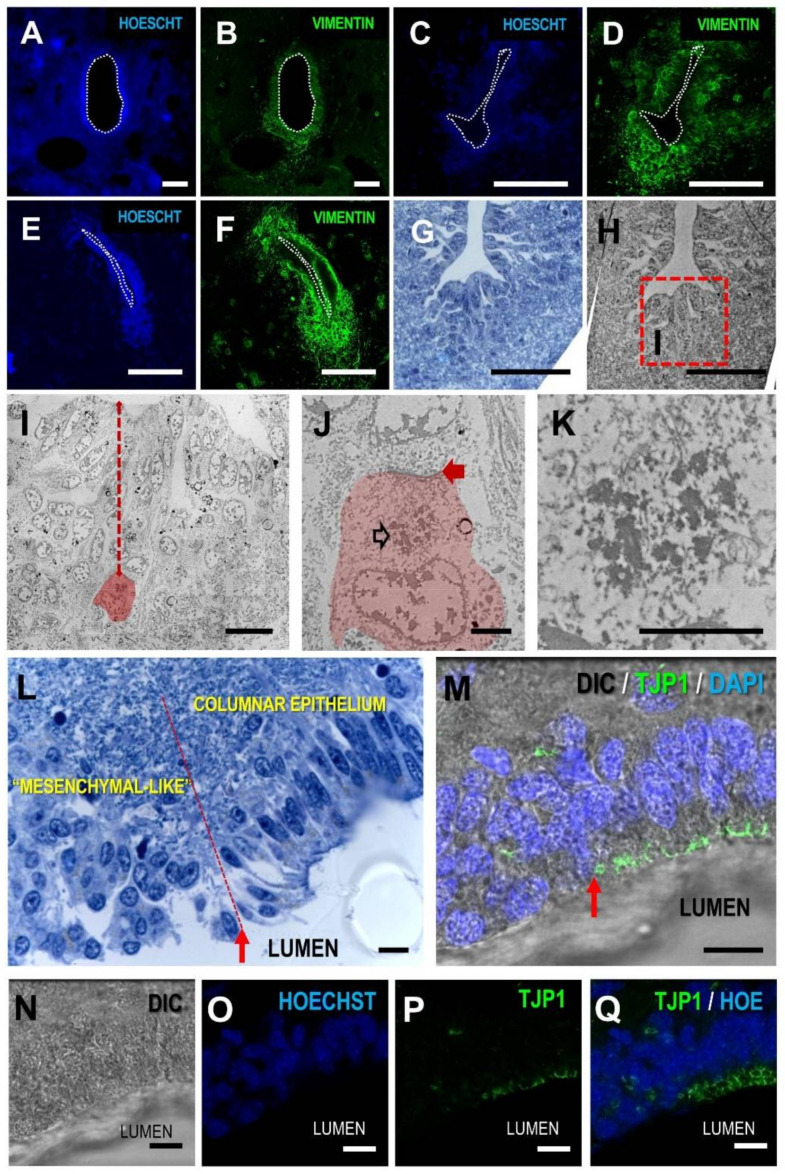
Cell delamination and loss of apical polarity are observed in human samples with a partially closed canal. (**A**,**B**) patent canal from a young individual *(BC01495 sample)* showing ependymal lining around central canal (nuclei blue, vimentin green); (**C**–**F**) partially patent canals with cell accumulation mainly in the ventral domains *(BC00654*, *BC00692 samples)*; (**G**) Toluidine blue 2 µm semithin section and its electron microscopy correlate obtained from *BC01800* sample; (**H–K**) shows abnormal accumulation of cells in the ventral domain with ectopic ependymal cells (red cell in **I**, arrow indicates distance from the central canal), in which cell junctional complexes (red arrow in **J**) and basal bodies from multiple cilia (empty arrow in **J**, detail in **K**) can be found; (**L**) lateral extension of a delaminating ependymal region in *BC01684* sample, showing transition from a columnar epithelium to a mesenchymal-like phenotype of adjacent cells (indicated by red arrow and dashed line); (**M**–**P**) this transition is accompanied by apico-basal cell polarity loss, including disassembling and loss of the apical marker TJP1 protein; (**Q**) maximal projection of confocal images stack show the transition between TJP1 apical distribution (green polygonal shapes) in the remaining ependymal lining and the sparse TJP1 staining in the delaminated domain. DIC, differential interference contrast, TJP1, Tight Junction Protein ZO1. Magnification bars: (**A**–**F**), 100 µm (**G**,**H**), 50 µm; (**I**,**L**–**Q**), 10 µm; (**J**,**K**), 2 µm.

After substantiating that the structure substituting central canal in humans is vastly different from that of other animals that spontaneously lose it, we aimed to collect evidence on the possible mechanisms underlying this specific process that results in unique human features. For this, we studied rare human spinal cord samples that are at different stages of the central canal closure instead of the normal samples lacking the central canal. We studied slices with canal patency (very rare event; Figure 5A,B) and slices with domains depicting abnormal cell accumulation or partial closure of the canal (Figure 5C–F).

In samples with a partially closed canal, we observed a general pattern that involved accumulation of cells mainly in the ventral domains, and the presence of ectopic ependymocytes found at long distances from the lumen (Figure 5G–K). In some domains, ependymal delamination is observed after losing apico-basal polarity and the loss of polarity markers such as Tight Junction Protein ZO-1 (TJP1) (Figure 5L–Q).

**Figure 6:**
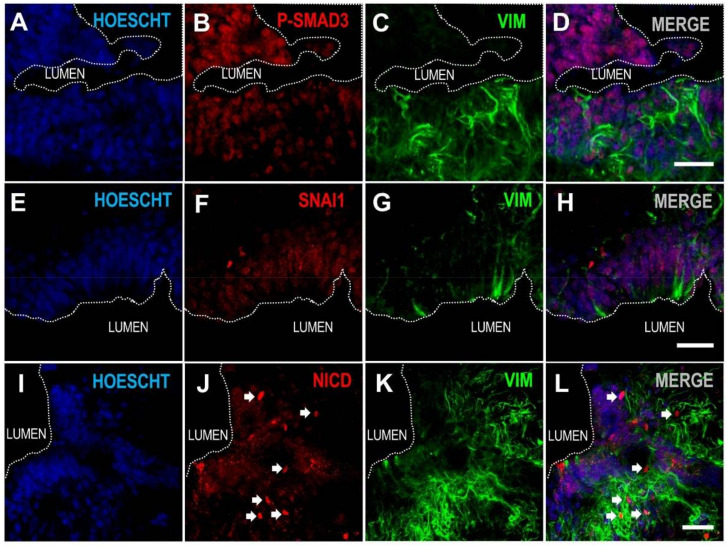
Expression of epithelial to mesenchymal related transcription factors in human delaminating ependyma. (**A**–**D**) ependymal cells in delaminating domains show nuclear expression of phosphorilated SMAD3; (**E**–**H**) Snai1; (**I**–**L**) and Notch Intracellular Domain (NICD, arrows). Magnification bars: 30 µm.

Ependymal delamination and loss of apico-basal polarity apparently involved the acquisition of a mesenchymal-like phenotype and included the nuclear expression of mediators related to epithelial to mesenchymal transitions such as the phosphorylated form of Mothers against decapentaplegic homolog 3 (SMAD3; Figure 6A–D), Zinc finger protein SNAI1 (SNAI1; Figure 6E–H), and the Notch Intracellular Domain (NICD; Figure 6I–L).

## 4. Discussion

The absence of the central canal in humans is a phenomenon already described in old manuals [56,57,58] but almost completely neglected afterwards, except for a few reports that deepen into that singularity [5,19,20,39,40,41,59,60]. This disregard by the scientific community may explain the current absence of animal models to study the process of central canal loss and the ignorance of how this unique human ependymal region impacts physiology and responses to spinal cord damage.

An approach to tackle this deficit could be the search for natural models in which animals spontaneously lose the central canal. After a thorough literature search, we found the Naked Mole-Rat (NMR) as candidate, a long-living rodent increasingly studied for its important particularities in the fields of aging, pain, cancer or social behavior [61,62,63,64]. We describe here for the first time that NMR indeed lack the central canal as adults, but this is replaced by a new organization of lamina X largely different from that in humans. This may suggest that the cause and/or the process of central canal loss could be different between NMR and humans. In the literature, we also found reports from other mammals such as porpoises [65,66], whales [67] and dolphins [68] describing an absence of central canal. Unfortunately, we could not obtain tissue samples for a further study, but the appearance of the lamina X in these species, according to the published images, looks similar to what we found in NMR, also separating them from the human case.

A few other mammals in which the central canal is absent are transgenic or mutant strains of laboratory mice. One of them, the hyh mice studied here, present a mutant variant of alpha-Snap protein, and show spinal cord central canal loss during late embryonic stages [48]. These mice suffer a continuing denudation of the ependymal lining that finally affects the aqueduct, leading to massive hydrocephalus and death during the first month of age [69,70]. This is indeed an important difference with humans (and NMR), since the loss of central canal in humans is a general feature in almost every individual, and the incidence of postnatal hydrocephaly in the general population is extremely low. When studying the central gray matter of adult hyh mice, we found that the canal is not replaced by a structure similar to humans. The histological features are much closer to NMR, although cell accumulations in the midline are not as clearly aligned as in NMR and some pseudocanals can be found, in contrast to NMR.

Other mutant mice in which the central canal disassembles or is completely lost are afadin mutant mice [71], Sox9 overexpressing mice [72] or mice with deletion of the Rho family guanosine triphosphatase (GTPase) 3 (Rnd3) [73]. Interestingly, many of these transgenic mice have in common the malfunction of cell adhesion related molecules, but none of them give rise to a human-like structure.

A different approach that would help to model human central canal closure is the experimental intervention to induce canal disassembling. There is only one such attempt published to date [40], using reovirus type 1 infection in rats for inducing ependymal damage, proliferation and canal closure. This strategy followed the rationale of the known tropism of viruses for ependymal cells, and the strong link between virus infection, inflammation and ependymal disassembling [74,75,76]. However, in all those reports, including the model by Milhorat et al. [77], severe complications were observed, mostly hydrocephaly, that are not found after human central canal loss. Moreover, the histological findings in [77] included the presence of pseudocanals, mild cellular accumulation and moderate gliosis, in a much lesser extent than those observed in humans.

Milhorat et al. hypothesize, based on their model, that “stenosis of the central canal in man is a pathological lesion involving ependymal injury and scarring”, but this may require some nuance. First, the sole lesion or mechanical damage to the ependymal cells is not enough to explain central canal de-structuring, since this does not happen after spinal cord injury in experimental animal models. In rodents, for example, when the ependymal region is damaged, it proliferates and contributes to the production of new ependymal cells and, in a lesser extent, to glial scar [12,14,78,79], but this does not involve a disassembling of the remaining canal neither rostral nor caudal to the injury site. In addition, the previous descriptions of human ependymal region [5,20,39,40,41] and the evidence we present here unveil a complex process that suggest an active delamination and transformation of the ependymal lining (induced by still unknown triggers), that ends up in a structure that includes astroglial reactivity, but is not limited to this, nor forms a proper scar.

What we observe here, after studying spinal cord levels with intermediate features between the fully patent canal and the total absence of canal, suggests that the process may probably begin with a ventral delamination of ependymal cells that may further extend to other aspects of the ependymal layer accompanied by the expression of factors involved in epithelial to mesenchymal transition, such as SNAI1, TGFb mediators (phospho-Smad3) and Notch signaling (NICD). Due to the low incidence of spinal ependymal tumors in humans [80], the epithelial to mesenchymal transition-like process that may underlie human central canal transformation, might probably reflect a transition to a fibrosis-like state (with gliosis), rather than to cancer formation [81,82,83] or to stemness [17]. Interestingly, EMT has been recently proposed to underlie the process of regeneration after spinal cord injury in zebrafish [84]. On the other hand, since the proliferation in the human ependymal region is rather low [5,20,35], the large accumulation of cells observed in adult humans at this region may also reflect a modulation of cell death, for which some of these factors, such as SNAI1 have been shown to be inhibitors [85].

The observation of ventral accumulation of ectopic ependymal cells can be observed in previous reports [35], and the delamination of ventral aspects (floor plate) is a phenomenon described in the normal formation of the ependymal lining during mammal development [86]. The involvement of factors such as Arx or Foxa2, specifically present in the ventral regions of the ependymal layer in the cases where it maintains patency, might be a topic to be explored in the future [6]. It must be also considered that other multiple steps and factors have been described in the normal formation of the central canal in rodents, in mechanisms that enables central canal formation without loss of ventricular layer integrity, including dorsal attrition, dorsal delamination of progenitors and a crucial role for Protein crumbs homolog 2 (Crb2) [86,87,88]. Whether some of these processes and factors are affected in animals spontaneously losing the central canal, or if they may be related to different programs for central canal disappearance that may explain differences between humans and the rest of the mammals, should be approached in future studies.

In conclusion, data from past reports and from this one may warrant caution when considering direct translation of the properties of the spinal cord ependymal region between species. It was already known that important differences in the identity, function and responses of ependymal cells in the spinal cord exist between regenerating (zebrafish) and non-regenerating animals [18] and even among species that regenerate, such as lizards and salamanders [89]. We suggest here that the adult human spinal cord is unique in this specific trait from the infancy, and shows vast morphological differences even with the few mammals that spontaneously lose central canal patency during their lifetime (NMR, cetaceans, mutant mice). These morphological and structural dissimilarities are also accompanied by genomic [6,19,20,60] and functional [7,35,42,90,91] differences. In order to successfully tackle the many unsolved issues in this field, (what is the trigger of the central canal loss in humans, the consequences of having this structure in the physiology of the cord and its response to damage…), it would be desirable to achieve experimental models that better mimic the human situation in the future.

## Figures and Tables

**Table 1:** Postmortem Human Spinal Cord tissue samples used for histology.

Autopsy Number	Cause of Death	Gender	Age	Canal Patency	Postmortem Delay
BC00659	Diffuse anoxia/ischemia	Male	48	Partially patent	Unknown
BC00692	Microhemorrhage areas in Brain	Male	19	Partially patent	Unknown
BC01495	Brain Edema	Male	14	Patent	Unknown
BC01684	Acute Hypoxia–ischemia	Male	27	Partially patent	Unknown
BC01736	Small Vessel Disease	Male	30	Patent	Unknown
BC01775	Acute Hypoxia–ischemia	Male	45	Partially patent	Unknown
BC01800	Hepatic encephalopathy	Male	56	Partially patent	Unknown
A07/044	Cardiopulmonary arrest	Male	39	Closed	3 h 30 min
A07/067	Refractory septic shock and cardiac arrest. Ischemic cardiopathy	Male	47	Closed	4 h 55 min
A10/017	Hepatic metastasis. Probable pancreatic neoplasia	Male	52	Closed	3 h

**Table 2:** List of Primary antibodies used.

Antibody	Host Species	Manufacturer	Catalogue Number	Dilution
Activated Notch1-NICD	Rabbit	Abcam	Ab8925	1:1000
GFAP	Chicken	Aves Labs	#GFAP	1:300
GFAP	Rabbit	DakoCytomation	#Z 0334	1:2000
Iba1	Rabbit	WAKO	019-19741	1:500
Laminin	Chicken	SIGMA	GW20044F	1:600
Nestin	Rabbit	ACRIS	APO9573PU-M	1:250
Phospho-SMAD3 (S423–S425)	Mouse	Abcam, clone EP823Y	Ab52903	1:100
S100β	Mouse	SIGMA-Aldrich, clone SH-B1	S2532	1:300
Snai1	Rabbit	Acris	AP20370PU-N	1:300
Sox2	Mouse	R&D Systems, clone 245610	MAB2018	1:200
Sox9	Goat	R&D Systems	AF3075	1:1000
TJP1 (ZO-1)	Mouse	Thermo, clone ZO1-1A12	33-9100	1:200
Vimentin	Mouse	DAKO clone V9	M0725	1:500

**Table 3:** List of Secondary antibodies used.

Antibody	Supplier	Dilution
Donkey anti-mouse biotin	Jackson IR, #715-065-151	1:500
Donkey anti-rabbit biotin	Jackson IR, #711-065-152	1:500
Streptavidin-Alexa 488	Jackson IR, #016-540-084	1:500
Donkey anti-mouse IgG Alexa 488	Invitrogen #A-21202	1:1000
Donkey anti-rabbit IgG Alexa 555	Invitrogen #A-31572	1:1000
Donkey anti-chicken IgY Cy5	Jackson IR, #703-175-155	1:500
Donkey anti-goat IgG Alexa 633	Invitrogen #A-21082	1:1000

## Data Availability

No new data were created or analyzed in this study apart from those presented in the manuscript. Data sharing is not applicable to this article.
